# 2-(1,3-Benzothia­zol-2-yl)guanidinium chloride

**DOI:** 10.1107/S1600536811044643

**Published:** 2011-10-29

**Authors:** Shaaban K. Mohamed, Peter N. Horton, Mahmoud A.A. El-Remaily, Seik Weng Ng

**Affiliations:** a Chemistry and Environmental Division, Manchester Metropolitan University, Manchester M15 6BH, England; b School of Chemistry, University of Southampton, Southampton SO17 1BJ, England; c Department of Chemistry, Faculty of Science, Sohag University, Egypt; d Department of Chemistry, University of Malaya, 50603 Kuala Lumpur, Malaysia; e Chemistry Department, King Abdulaziz University, PO Box 80203 Jeddah, Saudi Arabia

## Abstract

The non-H atoms of the cation of the title salt, C_8_H_9_N_4_S^+^·Cl^−^, are approximately co-planar (r.m.s. deviation = 0.037 Å), with one amino group forming an intra­molecular hydrogen bond to the tertiary N atom of the benzothia­zole fused-ring system. The cations and anions are linked by cyclic *R*_2_^1^(6) N—H⋯Cl hydrogen-bonding associations, generating helical chains running along the *b*-axis direction.

## Related literature

For the synthesis, see: Takahashi & Niino (1943 ▶). For the structure of 2-(1,3-benzothia­zol-2-yl)guanidine, see: Mohamed *et al.* (2011 ▶). For graph-set analysis, see: Etter *et al.* (1990 ▶).
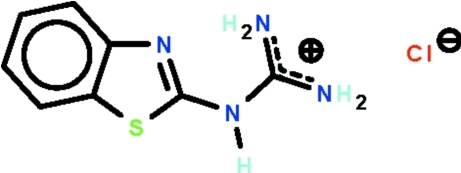


## Experimental

### 

#### Crystal data

C_8_H_9_N_4_S^+^·Cl^−^
*M* *_r_* = 228.71
Orthorhombic, 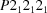

*a* = 3.8857 (5) Å
*b* = 11.0349 (17) Å
*c* = 22.186 (3) Å
*V* = 951.3 (2) Å^3^
*Z* = 4
Mo *K*α radiation
μ = 0.58 mm^−1^
*T* = 120 K
0.12 × 0.03 × 0.01 mm

#### Data collection

Rigaku Saturn 724+ diffractometer
Absorption correction: multi-scan (*CrystalClear*; Rigaku, 2011 ▶) *T*_min_ = 0.933, *T*_max_ = 0.994
14016 measured reflections
2146 independent reflections
2117 reflections with *I* > 2σ(*I*)
*R*_int_ = 0.034

#### Refinement

*R*[*F* ^2^ > 2σ(*F* ^2^)] = 0.030
*wR*(*F* ^2^) = 0.076
*S* = 1.07
2146 reflections
147 parameters
5 restraints
H atoms treated by a mixture of independent and constrained refinement
Δρ_max_ = 0.26 e Å^−3^
Δρ_min_ = −0.27 e Å^−3^
Absolute structure: Flack (1983 ▶), 839 Friedel pairs
Flack parameter: −0.01 (7)

### 

Data collection: *CrystalClear* (Rigaku, 2011 ▶); cell refinement: *CrystalClear*; data reduction: *CrystalClear*; program(s) used to solve structure: *SHELXS97* (Sheldrick, 2008 ▶); program(s) used to refine structure: *SHELXL97* (Sheldrick, 2008 ▶); molecular graphics: *X-SEED* (Barbour, 2001 ▶); software used to prepare material for publication: *publCIF* (Westrip, 2010 ▶).

## Supplementary Material

Crystal structure: contains datablock(s) global, I. DOI: 10.1107/S1600536811044643/zs2157sup1.cif

Structure factors: contains datablock(s) I. DOI: 10.1107/S1600536811044643/zs2157Isup2.hkl

Supplementary material file. DOI: 10.1107/S1600536811044643/zs2157Isup3.cml

Additional supplementary materials:  crystallographic information; 3D view; checkCIF report

## Figures and Tables

**Table 1:** Hydrogen-bond geometry (Å, °)

*D*—H⋯*A*	*D*—H	H⋯*A*	*D*⋯*A*	*D*—H⋯*A*
N2—H1⋯Cl1^i^	0.88 (1)	2.21 (1)	3.074 (2)	165 (2)
N3—H2⋯Cl1	0.88 (1)	2.62 (2)	3.380 (2)	146 (2)
N4—H3⋯Cl1	0.88 (1)	2.31 (1)	3.157 (2)	160 (2)
N4—H4⋯N1	0.88 (1)	2.06 (2)	2.713 (2)	131 (2)

Symmetry code: (i) 
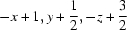
.

**Fig. 1.**
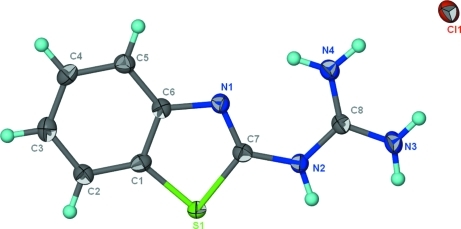
Thermal ellipsoid plot (Barbour, 2001) of C8H9N4S+ Cl- at the 70% probability level. Hydrogen atoms are drawn as spheres of arbitrary radius.

## supplementary crystallographic information

### Comment

A recent study (Mohamed *et al.*, 2011) describes the crystal structure of 2-(1,3-benzothiazol-2-yl)guanidine, which was synthesized by the reaction of 2-aminothiophenol and cyanoguanidine in 10% sulfuric acid medium. The product of the reaction is probably a sulfate or bisulfate salt that is then converted to the neutral compound upon treatment with sodium hydroxide. In the present study, 2-(1,3-benzothioazol-2-yl)guanidine is converted to the hydrochloride salt by treatment with hydrochloric acid. The non-H atoms of the cation of the title salt, C_8_H_9_N_4_S^+^ Cl^-^ (Scheme I), lie on a plane (r.m.s. deviation 0.037 Å), with one amino group forming an intramolecular hydrogen bond to the tertiary N atom of the benzothiazole fused-ring (Fig. 1). The cations and anions are linked by cyclic N—H···Cl hydrogen-bonding associations [graph set *R*^1^_2_(6) (Etter *et al.*, 1990)] (Table 1), to generate helical chains running along the *b*-axis of the orthorhombic unit cell. This salt was first reported in 1943 (Takahashi & Niino, 1943).

### Experimental

2-(1,3-Benzothiazol-2-yl)guanidine (0.05 mol) was heated in ethanol (50 ml) in the presence of a few drops of hydrochloric acid for 3 h. The mixture was cooled and the product was collected and recrystallized from ethanol to give the title compound (m.p. 523 K) in 95% yield; .

### Refinement

Carbon-bound H-atoms were placed in calculated positions (C—H = 0.95 Å) and were included in the refinement in the riding model approximation, with *U*_iso_(H) set to 1.2*U*_eq_(C). The amino H-atoms were located in a difference Fourier map, and were refined with a distance restraint of N—H = 0.88±0.01 Å with their isotropic displacement parameters freely refining.

### Crystal data

**Table:**

C_8_H_9_N_4_S^+^·Cl^−^	*D*_x_ = 1.597 Mg m^−^^3^
*M**_r_* = 228.71	Melting point: 523 K
Orthorhombic, *P*2_1_2_1_2_1_	Mo *K*α radiation, λ = 0.71073 Å
Hall symbol: P 2ac 2ab	Cell parameters from 3321 reflections
*a* = 3.8857 (5) Å	θ = 2.1–31.0°
*b* = 11.0349 (17) Å	µ = 0.58 mm^−^^1^
*c* = 22.186 (3) Å	*T* = 120 K
*V* = 951.3 (2) Å^3^	Lath, colorless
*Z* = 4	0.12 × 0.03 × 0.01 mm
*F*(000) = 472	

### Data collection

**Table:**

Rigaku Saturn 724+ diffractometer	2146 independent reflections
Radiation source: rotating anode	2117 reflections with *I* > 2σ(*I*)
confocal	*R*_int_ = 0.034
Detector resolution: 28.5714 pixels mm^-1^	θ_max_ = 27.5°, θ_min_ = 2.1°
ω scans	*h* = −4→4
Absorption correction: multi-scan (*CrystalClear*; Rigaku, 2011)	*k* = −14→14
*T*_min_ = 0.933, *T*_max_ = 0.994	*l* = −28→28
14016 measured reflections	

### Refinement

**Table:**

Refinement on *F*^2^	Secondary atom site location: difference Fourier map
Least-squares matrix: full	Hydrogen site location: inferred from neighbouring sites
*R*[*F*^2^ > 2σ(*F*^2^)] = 0.030	H atoms treated by a mixture of independent and constrained refinement
*wR*(*F*^2^) = 0.076	*w* = 1/[σ^2^(*F*_o_^2^) + (0.0454*P*)^2^ + 0.3169*P*] where *P* = (*F*_o_^2^ + 2*F*_c_^2^)/3
*S* = 1.07	(Δ/σ)_max_ = 0.001
2146 reflections	Δρ_max_ = 0.26 e Å^−^^3^
147 parameters	Δρ_min_ = −0.27 e Å^−^^3^
5 restraints	Absolute structure: Flack (1983), 839 Friedel pairs
Primary atom site location: structure-invariant direct methods	Flack parameter: −0.01 (7)

### Special details

**Table:**

Geometry. All e.s.d.'s (except the e.s.d. in the dihedral angle between two l.s. planes) are estimated using the full covariance matrix. The cell e.s.d.'s are taken into account individually in the estimation of e.s.d.'s in distances, angles and torsion angles; correlations between e.s.d.'s in cell parameters are only used when they are defined by crystal symmetry. An approximate (isotropic) treatment of cell e.s.d.'s is used for estimating e.s.d.'s involving l.s. planes.

### Fractional atomic coordinates and isotropic or equivalent isotropic displacement parameters (Å^2^)

**Table:**

	*x*	*y*	*z*	*U*_iso_*/*U*_eq_	
Cl1	0.94400 (14)	0.19472 (4)	0.72036 (2)	0.02120 (13)	
S1	0.32449 (13)	0.63436 (4)	0.96176 (2)	0.01619 (12)	
N1	0.6155 (4)	0.42234 (14)	0.95141 (7)	0.0168 (4)	
N2	0.4500 (5)	0.51363 (15)	0.85919 (7)	0.0175 (4)	
H1	0.344 (7)	0.5752 (17)	0.8420 (12)	0.032 (7)*	
N3	0.4832 (5)	0.43982 (16)	0.76315 (7)	0.0209 (4)	
H2	0.539 (7)	0.3806 (17)	0.7388 (10)	0.027 (7)*	
H5	0.390 (8)	0.5097 (17)	0.7530 (14)	0.053 (10)*	
N4	0.7193 (5)	0.32801 (15)	0.83996 (8)	0.0211 (4)	
H3	0.792 (7)	0.2752 (18)	0.8130 (9)	0.029 (7)*	
H4	0.766 (7)	0.320 (2)	0.8786 (5)	0.023 (6)*	
C1	0.4490 (5)	0.55994 (17)	1.02742 (9)	0.0168 (4)	
C2	0.4162 (5)	0.59888 (18)	1.08674 (9)	0.0179 (4)	
H2A	0.3142	0.6748	1.0962	0.021*	
C3	0.5382 (5)	0.52252 (18)	1.13174 (9)	0.0195 (4)	
H3A	0.5207	0.5467	1.1727	0.023*	
C4	0.6856 (6)	0.41107 (18)	1.11753 (9)	0.0188 (4)	
H4A	0.7657	0.3602	1.1491	0.023*	
C5	0.7178 (6)	0.37284 (18)	1.05846 (9)	0.0186 (4)	
H5A	0.8187	0.2967	1.0492	0.022*	
C6	0.5993 (5)	0.44847 (17)	1.01311 (9)	0.0160 (4)	
C7	0.4809 (5)	0.51068 (17)	0.92100 (9)	0.0158 (4)	
C8	0.5560 (6)	0.42508 (17)	0.82086 (8)	0.0162 (4)	

### Atomic displacement parameters (Å^2^)

**Table:**

	*U*^11^	*U*^22^	*U*^33^	*U*^12^	*U*^13^	*U*^23^
Cl1	0.0242 (3)	0.0190 (2)	0.0205 (2)	−0.0008 (2)	−0.00028 (19)	−0.00403 (19)
S1	0.0198 (2)	0.0132 (2)	0.0156 (2)	0.00165 (17)	−0.0007 (2)	0.00035 (18)
N1	0.0209 (9)	0.0139 (7)	0.0155 (8)	−0.0013 (6)	−0.0004 (7)	−0.0005 (6)
N2	0.0254 (9)	0.0127 (7)	0.0144 (8)	0.0006 (7)	−0.0006 (7)	0.0001 (6)
N3	0.0310 (10)	0.0157 (8)	0.0159 (8)	−0.0017 (8)	−0.0003 (8)	−0.0036 (7)
N4	0.0290 (10)	0.0167 (8)	0.0174 (8)	0.0022 (8)	0.0016 (8)	−0.0009 (7)
C1	0.0172 (9)	0.0141 (8)	0.0191 (9)	−0.0010 (8)	−0.0012 (8)	0.0029 (7)
C2	0.0183 (10)	0.0164 (9)	0.0189 (9)	−0.0014 (8)	0.0021 (8)	−0.0008 (7)
C3	0.0190 (10)	0.0228 (10)	0.0167 (9)	−0.0059 (9)	0.0013 (8)	0.0004 (8)
C4	0.0181 (9)	0.0204 (9)	0.0178 (9)	−0.0017 (9)	−0.0013 (8)	0.0053 (7)
C5	0.0205 (10)	0.0155 (9)	0.0199 (9)	0.0007 (8)	0.0004 (8)	0.0007 (7)
C6	0.0158 (10)	0.0154 (9)	0.0169 (9)	−0.0022 (7)	0.0009 (7)	0.0000 (7)
C7	0.0161 (10)	0.0134 (8)	0.0179 (9)	−0.0015 (7)	0.0011 (8)	0.0006 (7)
C8	0.0191 (9)	0.0142 (8)	0.0154 (9)	−0.0024 (8)	0.0033 (8)	0.0008 (7)

### Geometric parameters (Å, °)

**Table:**

S1—C1	1.7409 (19)	N4—H4	0.882 (10)
S1—C7	1.746 (2)	C1—C2	1.390 (3)
N1—C7	1.296 (2)	C1—C6	1.398 (3)
N1—C6	1.400 (2)	C2—C3	1.390 (3)
N2—C8	1.359 (2)	C2—H2A	0.9500
N2—C7	1.377 (2)	C3—C4	1.393 (3)
N2—H1	0.882 (10)	C3—H3A	0.9500
N3—C8	1.321 (3)	C4—C5	1.382 (3)
N3—H2	0.876 (10)	C4—H4A	0.9500
N3—H5	0.880 (10)	C5—C6	1.386 (3)
N4—C8	1.315 (3)	C5—H5A	0.9500
N4—H3	0.883 (10)		
			
C1—S1—C7	88.16 (9)	C2—C3—H3A	119.6
C7—N1—C6	109.63 (17)	C4—C3—H3A	119.6
C8—N2—C7	125.43 (17)	C5—C4—C3	121.41 (19)
C8—N2—H1	115.2 (19)	C5—C4—H4A	119.3
C7—N2—H1	119.3 (19)	C3—C4—H4A	119.3
C8—N3—H2	116.9 (18)	C4—C5—C6	118.31 (19)
C8—N3—H5	116 (2)	C4—C5—H5A	120.8
H2—N3—H5	127 (3)	C6—C5—H5A	120.8
C8—N4—H3	118.3 (16)	C5—C6—C1	120.25 (18)
C8—N4—H4	119.6 (16)	C5—C6—N1	124.80 (18)
H3—N4—H4	122 (2)	C1—C6—N1	114.95 (17)
C2—C1—C6	121.68 (18)	N1—C7—N2	124.85 (18)
C2—C1—S1	128.38 (15)	N1—C7—S1	117.31 (15)
C6—C1—S1	109.95 (14)	N2—C7—S1	117.84 (14)
C3—C2—C1	117.47 (18)	N4—C8—N3	121.06 (18)
C3—C2—H2A	121.3	N4—C8—N2	122.02 (18)
C1—C2—H2A	121.3	N3—C8—N2	116.92 (18)
C2—C3—C4	120.87 (19)		
			
C7—S1—C1—C2	179.9 (2)	S1—C1—C6—N1	−0.1 (2)
C7—S1—C1—C6	0.28 (16)	C7—N1—C6—C5	179.4 (2)
C6—C1—C2—C3	−0.1 (3)	C7—N1—C6—C1	−0.3 (2)
S1—C1—C2—C3	−179.68 (17)	C6—N1—C7—N2	−178.94 (19)
C1—C2—C3—C4	−0.3 (3)	C6—N1—C7—S1	0.5 (2)
C2—C3—C4—C5	0.4 (3)	C8—N2—C7—N1	−0.2 (3)
C3—C4—C5—C6	0.0 (3)	C8—N2—C7—S1	−179.64 (17)
C4—C5—C6—C1	−0.5 (3)	C1—S1—C7—N1	−0.50 (16)
C4—C5—C6—N1	179.77 (19)	C1—S1—C7—N2	179.02 (17)
C2—C1—C6—C5	0.6 (3)	C7—N2—C8—N4	−3.7 (3)
S1—C1—C6—C5	−179.81 (16)	C7—N2—C8—N3	175.3 (2)
C2—C1—C6—N1	−179.68 (18)		

### Hydrogen-bond geometry (Å, °)

**Table:**

*D*—H···*A*	*D*—H	H···*A*	*D*···*A*	*D*—H···*A*
N2—H1···Cl1^i^	0.88 (1)	2.21 (1)	3.074 (2)	165 (2)
N3—H2···Cl1	0.88 (1)	2.62 (2)	3.380 (2)	146 (2)
N4—H3···Cl1	0.88 (1)	2.31 (1)	3.157 (2)	160 (2)
N4—H4···N1	0.88 (1)	2.06 (2)	2.713 (2)	131 (2)

Symmetry codes: (i) −*x*+1, *y*+1/2, −*z*+3/2.
